# Effect of Nitrogen Doping on the Crystallization Kinetics of Ge_2_Sb_2_Te_5_

**DOI:** 10.3390/nano11071729

**Published:** 2021-06-30

**Authors:** Minh Anh Luong, Nikolay Cherkashin, Béatrice Pecassou, Chiara Sabbione, Frédéric Mazen, Alain Claverie

**Affiliations:** 1 CEMES-CNRS, 29 Rue Jeanne Marvig, 31055 Toulouse, France; minh-anh.luong@cemes.fr (M.A.L.); nikolay.cherkashin@cemes.fr (N.C.); beatrice.pecassou@cemes.fr (B.P.); 2 Léti-CEA, 17 Avenue des Martyrs, F-38000 Grenoble, France; chiara.sabbione@cea.fr (C.S.); frederic.mazen@cea.fr (F.M.)

**Keywords:** phase change materials, Ge_2_Sb_2_Te_5_, nitrogen, crystallization, strain, kinetics

## Abstract

Among the phase change materials, Ge_2_Sb_2_Te_5_ (GST-225) is the most studied and is already integrated into many devices. N doping is known to significantly improve some key characteristics such as the thermal stability of materials and the resistance drift of devices. However, the origin, at the atomic scale, of these alterations is rather elusive. The most important issue is to understand how N doping affects the crystallization characteristics, mechanisms and kinetics, of GST-225. Here, we report the results of a combination of in situ and ex situ transmission electron microscopy (TEM) investigations carried out on specifically designed samples to evidence the influence of N concentration on the crystallization kinetics and resulting morphology of the alloy. Beyond the known shift of the crystallization temperature and the observation of smaller grains, we show that N renders the crystallization process more “nucleation dominated” and ascribe this characteristic to the increased viscosity of the amorphous state. This increased viscosity is linked to the mechanical rigidity and the reduced diffusivity resulting from the formation of Ge–N bonds in the amorphous phase. During thermal annealing, N hampers the coalescence of the crystalline grains and the cubic to hexagonal transition. Making use of AbStrain, a recently invented TEM-based technique, we evidence that the nanocrystals formed from the crystallization of N-doped amorphous GST-225 are under tension, which suggests that N is inserted in the lattice and explains why it is not found at grain boundaries. Globally, all these results demonstrate that the origin of the effect of N on the crystallization of GST-225 is not attributed to the formation of a secondary phase such as a nitride, but to the ability of N to bind to Ge in the amorphous and crystalline phases and to unbind and rebind with Ge along the diffusion path of this atomic species during annealing.

## 1. Introduction

Phase change materials (PCMs) are materials which show dramatic variations of several of their physical properties, such as the optical reflectance and electrical resistivity, which result from a change in their structure from amorphous to the crystalline states [1,2,3,4,5]. After their successful exploitation in compact disc read-only memories (CD ROMs), PCMs are currently exploited in phase change random-access memories (PC-RAMs), where the bit of information is encoded within two distinct resistive states corresponding to the (high-resistive) amorphous state and the (low-resistive) crystalline state. Reversible SET to RESET transitions are obtained by feeding a cell with appropriate pulses of electrical current and heating the material, generally a small dome of a few tens of nanometers in diameter, to crystallize it or, alternatively, to quench it from the melt. 

The high resistivity contrast and the fast switching between these states offered by Ge_2_Sb_2_Te_5_ (GST-225) have motivated its integration into high-performance digital devices [6,7]. Moreover, recent reports have demonstrated the possibility to program multilevel cells using GST-225, a promising step towards their integration as synaptic elements in artificial neural networks, as needed for neuromorphic computing [8,9,10]. 

However, GST-225 shows several characteristics which severely limits the application field of devices using it. Of first concern is its limited thermal stability. Its crystallization temperature (from 120 to 180 °C, depending on purity, homogeneity and layers in contact) is too low to preserve code integrity during soldering processes, as needed for embedded applications, and to ensure good data retention under moderate temperature conditions. Another drawback is the tendency of the resistivity of the RESET state to drift over time, probably the result of some structural relaxation of the amorphous phase obtained after quenching from the melt [11]. For these reasons, there is an increasing demand for PCMs exhibiting better thermal stability. 

Doping with chemical impurities may bring solutions. Carbon [12], oxygen [13], bismuth [14] and antimony [15] have been reported to increase the crystallization temperature of GST-225. Doping with a few percent of nitrogen is also appealing because it represents an effective way to achieve much higher crystallization temperatures (T_x_) [16,17,18,19], increased resistivity of both the crystalline and amorphous states while maintaining a high contrast [20,21,22,23,24], and a reduced resistivity drift of the RESET state [25,26]. Moreover, N has also been shown to render the transition from the amorphous to the crystalline states more progressive, giving more precise access to intermediate resistivity states between the RESET and SET values [21].

There are many reports on the effect of N on the thermal crystallization and resulting microstructure of GST-225. There is an overall consensus that T_x_ gradually increases with N concentration and that a maximum crystallization temperature of about 250 °C can be reached for concentrations in the 8–12% range [16,20,21,22,23,24,27,28,29,30,31]. However, for much higher concentrations, the Ge-poor Ge_1_Sb_2_Te_4_ phase crystallizes instead of the desired GST-225 [16]. Moreover, N doping also seems to shift the cubic to hexagonal transition to a much higher temperature, classically occurring at about 300 °C in pure GST-225 [16,29]. Another noticeable effect of N doping is a significant reduction in the size of the grains which are formed after crystallization [22,24,28,31].

Again, although these effects have been well established, their origin is unclear. As the thermal crystallization of GST-225 proceeds through different steps, incubation, nucleation and growth, one reason for this limited understanding lies in the difficulty to infer the mechanisms which are impacted by N during crystallization based on the sole observation of the microstructure of the material after annealing. Alternatively, Privitera et al. [23], while using resistivity measurements, a quite indirect technique, have shown that in situ measurements during annealing provide more insights into those mechanisms, revealing how their respective kinetics are impacted. Thus, we believe that the observation and recording of the full sequence of crystallization of GST-225 during in situ annealing in a transmission electron microscope (TEM) would be very much appropriate to pinpoint the impact of N on the nucleation and growth of the crystalline phase.

Another issue is that, most often and whatever the characterization technique, the influence of N is studied through the comparison of the results obtained on a limited number of layers doped at different N concentrations. If in situ annealing in the TEM is to be used, samples of different concentrations are to be annealed in situ one by one, rendering the extraction of reliable data for comparison, at best, delicate. To circumvent this difficulty, samples of doped and undoped GST-225 should be preferably annealed at the same time, during the same in situ annealing experiment. This can be achieved by preparing specifically designed layers by combining deposition and ion implantation techniques. Through the careful selection of layer thickness, ion beam energy and fluence, a reasonably thick layer consisting of a N-doped region on top a pristine amorphous GST-225 can be fabricated. Moreover, the concentration profile resulting from the ion implantation may be exploited to assess the influence of the concentration over a large range of values.

Given the remarks above, we have decided to combine the advantages provided by in situ annealing experiments in the TEM and these N-implanted GST-225 layers. This work reports the direct imaging of the crystallization sequences which affect amorphous GST-225 during annealing and evidences the morphological and kinetic differences due to N doping. This allows for a fact-based discussion on the underlying mechanisms.

The second point to clarify the atomic location of N. There is experimental evidence that, in the amorphous phase, N preferentially binds to Ge, eventually forming nitrides for high N concentrations [21,22,32]. In the crystalline phase, there is no consensus. Simulations tend to suggest that inserting N into crystalline GST-225 is too costly and that it will be expelled at crystallization [33]. This reinforced the widespread belief that N, in the form of nitrides, resides at the grain boundaries then inhibits the further growth of these grains [28,33,34,35]. However, one has to note that, although nitride-like characteristics have been readily evidenced in crystalline N-doped GST-225 [23,32], there are no reports showing TEM images or other direct experimental evidence of such phases decorating the GST-225 grain boundaries. On the contrary, there are several reports showing, by X-ray diffraction (XRD), the increase in the lattice parameters of polycrystalline GST-225 layers for increasing N concentrations. The interpretation of data differs, however, as well as the range of concentrations investigated, assigning this characteristic either to the occupation of vacancy sites [24] in the (Ge, Sb, V) sub-lattice or to the insertion of N into tetrahedral sites [28,30].

It is to be noted that all these results were obtained from a very limited number of samples, in terms of annealing conditions and N concentrations. They were also obtained on fully crystallized layers. Ideally, measurements aimed at evidencing the effect of N incorporation on the strain state of crystalline GST-225 should be carried out on single grains, before they come in contact and possibly exert stress on each other, i.e., in samples where the material is only partially crystallized. 

For this reason, we have used a recently invented TEM-based technique, named “AbStrain”, initially developed to correct experimental high resolution (HR) TEM images from distortions and calibration errors and for mapping the exact interplanar distances and angles in crystals with high precision [36]. Here, this technique is used to measure the changes of interplanar spacing (strain) of small GST-225 nanocrystals resulting from the eventual incorporation of N upon crystallization. 

Thanks to a unique combination of advanced techniques of TEM and specifically designed samples, in this paper we show that the incorporation of N renders the crystallization of GST-225 more dominated by nucleation, a characteristic to be ascribed, along with the much larger crystallization temperature, to the increased viscosity of the N-doped amorphous GST. Moreover, we evidence that small grains crystallizing in N-doped GST-225 show strong positive strain, hence suggesting the direct incorporation of N into the GST lattice.

## 2. Experimental

A 500 nm thick Ge_2_Sb_2_Te_5_ film was grown in the amorphous state by physical vapor deposition on a naturally oxidized 300 mm silicon (100) wafer using an industrial tool. The Ge_2_Sb_2_Te_5_ film was capped in situ with a 20 nm thick GeN layer to protect it from oxidation. The wafer was cut into pieces then implanted with nitrogen ions at 80 keV with a fluence of 3.8 × 10^16^ ions/cm^2^. These conditions were intended to incorporate a maximum concentration of about 5% (atomic fraction) at a depth of about 150 nm from the surface and a concentration decreasing down to almost 0% at the surface and at a depth of about 300 nm. A 200 nm thick bottom part of the 500 nm thick GST layer was thus left undoped, providing a reference in the same sample. Preliminary characterization of the as-implanted films has evidenced that ion implantation may cause the recrystallization of the layer, due to heating and collisional effects. For this reason, a very low beam current of 40 μA was used for implantation, for which the layer was checked to have remained fully amorphous (Supplementary Materials Figure S2). Subsequently, 1 × 1 cm^2^ specimens were annealed in a horizontal Carbolite furnace under atmospheric pressure and N_2_ gas flow for temperatures ranging from 170 to 300 °C and times from 30 min to 1 h. Thin samples of the annealed layers, suitable for cross-sectional transmission electron microscopy (XTEM) observations, were prepared by focus ion beam (FIB) using an FEI Helios NanoLab 600 (FEI Company) operating with a 30 keV Ga ion beam and finally polished and cleaned at 2 keV. The samples were imaged and analyzed by various TEM techniques using either an aberration-corrected FEI TECNAI F20 (200 KeV), a Philips CM20-FEG (200 KeV) or the I^2^TEM from Hitachi (300 KeV) [37]. 

**Figure 1:**
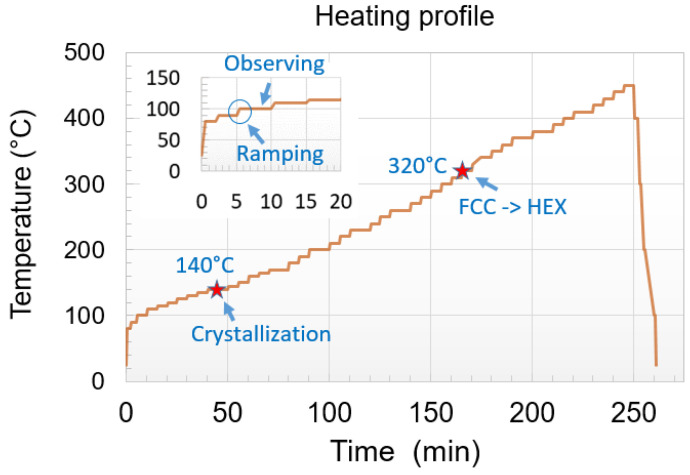
Typical heating profile used for in situ TEM annealing. The temperature was increased by steps of 5 °C from about 100 °C, using a ramping rate of 1 °C/s, and held for 5 min at this temperature during which the film was imaged (shown by the insertion). FCC to HEX refers to the transition from the face-centered cubic to the hexagonal phase.

In parallel, the thermal crystallization of the implanted and non-implanted films was observed during in situ annealing in a TEM, in bright-field and dark-field modes, and recorded. This in situ heating was performed using a Gatan 652 double tilt heating holder which was connected to a temperature controller and acted as a furnace-type holder. In the experiments presented here, the temperature was increased by steps of 5 °C from about 100 °C, using a ramping rate of 1 °C/s, and held for 5 min at this temperature during which the film was imaged (Figure 1). Special attention has been paid to reduce the influence of electron beam irradiation on the crystallization by minimizing the exposure time and beam intensity [29]. Moreover, fresh Ge_2_Sb_2_Te_5_ areas were also analyzed and compared to those left under the beam.

## 3. Results and Discussion

### 3.1. Crystallization Kinetics

#### 3.1.1. Pristine Sample

In situ TEM is a powerful technique, but artefacts may affect the kinetics and even the type of the observed phenomena, due to the limited specimen thickness, its possible oxidation, electron beam irradiation and heating effects. For this reason, before giving credit to the results obtained when studying the thermal behavior of N-implanted Ge_2_Sb_2_Te_5_ in situ, we first investigated the crystallization of pure and amorphous Ge_2_Sb_2_Te_5_ for referencing. The structure and stoichiometry of the as-deposited Ge_2_Sb_2_Te_5_ were checked by TEM and energy dispersive X-ray spectroscopy (EDX), and the results are shown in the Supplementary Materials Figure S1.

**Figure 2:**
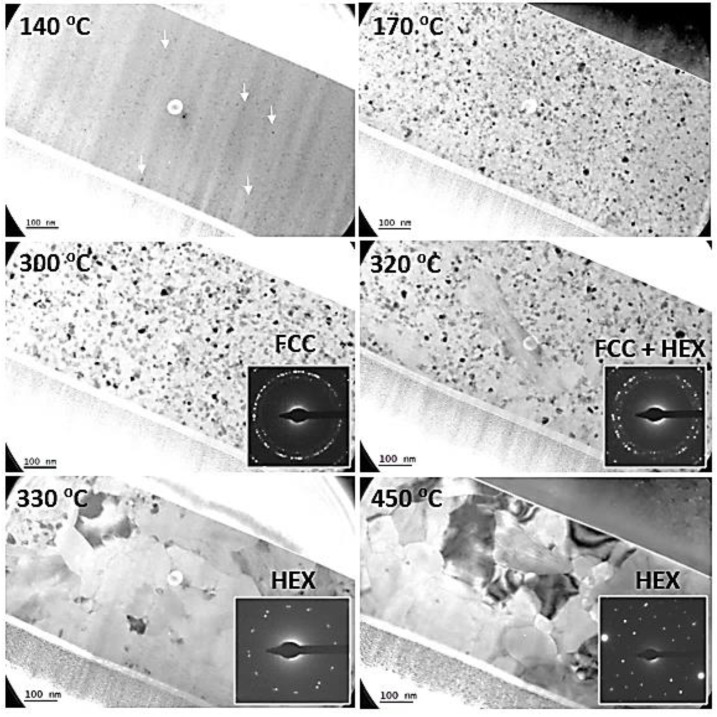
Bright-field (BF) TEM micrographs showing the structure of a pristine Ge_2_Sb_2_Te_5_ layer during the increase in the annealing temperature during the in situ experiment. The inserts show the associated SAED patterns. “FCC” and “HEX” refer to the face-centered cubic and hexagonal structures, respectively.

Figure 2 is a montage of snapshots summarizing the main results which were extracted from a video taken during the heating of the specimen. Up to 135 °C, nothing happened. At 140 °C, small crystals started to nucleate homogeneously within the layer (indicated by white arrows). They were small, typically from 2 to 8 nm in diameter (mean diameter of 5 nm). With increasing time and temperature, these grains grew while more grains nucleated.

From about 170 °C, i.e., after having spent about 30 min above 140 °C, the layer was, for a large part, crystalline, as deduced from the selected area electron diffraction (SAED) pattern and the diffraction contrast in the image. Beyond this temperature, the analysis of the grain population revealed that the grains continuously increased in size during ramping up (see Table 1), up to 300 °C. Electron diffraction patterns show the expected face-centered cubic (FCC) structure signature of the GST-225 grains. This regular and quite slow growth regime is thought to mostly result from the crystallization of the remaining amorphous material, although the competitive growth of existing nanocrystals may also contribute. However, from 300 °C and above, coalescence of the grains was observed, and consequently, the size of the grains dramatically increased. It is interesting to note that the new large grains which resulted from this coalescence showed the hexagonal stable structure of GST-225. From 330 °C and above, all grains were quite large (a few hundred nanometers) and showed a hexagonal structure. They did not evolve significantly in size but showed better defined grain boundaries when heated up to 450 °C (see Supplementary Materials Video S1–S4 for the real-time observation of the evolution of the structure). 

Thus, the information that we could extract from our in situ TEM observation of the crystallization of stoichiometric GST-225 was in very good agreement with those reported in the literature [2,3,4]. One could probably argue that the crystallization temperature that we observed, 10 to 20 °C below the most commonly reported values, may be due to the oxidation of the FIB specimen or to electron beam irradiation. Oxidation results in the heterogeneous and fast nucleation of the crystalline phase from the oxidized layer [38], a phenomenon which we have observed on intentionally oxidized layers but not in the experiments reported here. Electron irradiation may also facilitate the nucleation and growth of GST grains [29]. However, we have systematically compared images obtained from “fresh” and beam-exposed areas without detecting quantifiable differences, thanks to the experimental precautions under which the specimens were imaged. Instead, we prefer to stress that, usually, the crystallization temperature is identified through resistivity measurements during fast and continuous heating of the material, typically 10 °C/min. In our experiments, the heating rate was high (1 °C/s), but the sample was maintained at constant temperature for 5 min after every 5 °C jump. Thus, on average, more time was left for the material to incubate and nucleate the crystalline phase, which we think is the reason for it presenting a slightly lower crystallization temperature. 

However, this test-study demonstrates that, provided basic precautions are respected, the in situ heating of thin GST lamellas in a TEM is a reliable technique to investigate the details of the crystallization phenomenon.

#### 3.1.2. N-Implanted Samples

**Figure 3:**
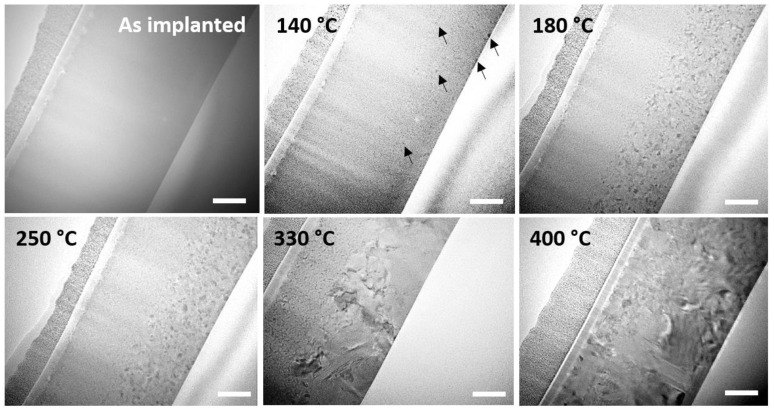
BF TEM micrographs showing the evolution of the structure of the N-implanted GST-225 layer during in situ annealing. The white scale bars refer to a length of 100 nm.

Figure 3 is a montage of snapshots showing the behavior and structural characteristics of the N-implanted layer during the same type of in situ annealing. From 140 °C, the nucleation of the crystalline phase is observed to start, as in the pristine sample, but only in the very surface region and at depths larger than 300 nm, i.e., in the regions where the N concentration is low, but not in the implanted region. When increasing the temperature up to 250 °C, these GST crystalline grains grew while the implanted region from below the surface towards a depth of approximately 300 nm remained amorphous. It was only when reaching 250 °C that the first crystalline nuclei started to appear in this region. At 330 °C, grains in the unimplanted bottom region dramatically grew and progressively transformed into the hexagonal phase. They even started growing from their initial location into the implanted region of the layer. Interestingly, the grains located in the doped region appeared to grow more slowly that those located in the bottom part. Finally, when reaching 400 °C, the layer was fully crystalline but still showed clear grain size differences depending on whether the grains sat in the N-doped or undoped regions. 

**Figure 4:**
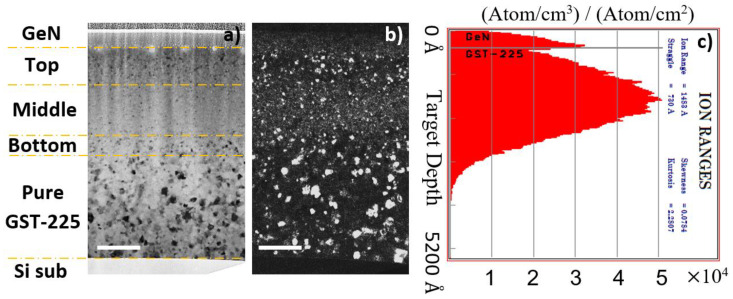
BF (**a**) and dark-field (DF) (**b**) images of the N-implanted GST-225 layer, annealed at 180 °C for 30 min. The N-implanted region is divided into different regions, the top, middle and bottom which are N-implanted, and the unimplanted region which provides the pristine reference. (**c**) SRIM simulation of the nitrogen depth-distribution after implantation. The white scale bars refer to a length of 100 nm.

To clarify the impact of N on the growth of the GST grains, we have measured their sizes in samples annealed ex situ under well-controlled conditions, which is more adapted and precise than from fast-evolving BF images taken during in situ annealing. Figure 4 shows, as a typical example, a set of BF and dark-field (DF) XTEM images obtained on the sample annealed at 180 °C for 30 min. The DF image clearly reveals that the grain sizes are distributed along the depth, in correspondence to the concentration of implanted nitrogen, as shown by the implanted profile (Figure 4c) extracted from the Monte Carlo simulation of the implantation [39]. 

To analyze such images, we divided the specimen into four regions: the top, extending from the GeN/GST interface to a depth of approximately 80 nm; the middle, from this depth to about 280 nm, i.e., centered on the projected range of the ions (Rp); the bottom, from this depth to about 320 nm; and the unimplanted region, which extended towards the substrate. 

Table 2 shows the results of the statistical analysis of the grain sizes in each of these regions, as a function of annealing conditions. 

In the unimplanted region, the grains had a mean size of about 18–22 nm after annealing at 170 °C for 30 min and did not grow further when increasing the annealing temperature until they coalesced at 300 °C and above, showing a sudden and dramatic increase in the grain size. In contrast, in the middle region, where the N concentration was above 2%, grain nucleation was not activated after 170 °C/30 min annealing and only started after annealing at 180 °C. Increasing the annealing temperature allowed for the growth of these grains only up to a size of about 10 nm, and did not evolve further even when annealing at 300 °C for 1 h. In the top and bottom regions, where the N concentration ranged from 1% to 2%, the situation was intermediate; the nucleation only started after 175 °C/30 min annealing and the grains further grew when increasing the temperature, but only up to a size of about 10 nm after 300 °C/1 h annealing. 

## 4. Discussion

The experimental results reported above show that the doping by N of amorphous GST-225 does not change its crystallization mechanism. Crystallization still proceeds through the homogeneous nucleation of grains followed by their growth. However, we have evidenced that, in the presence of N, the nucleation regime requires a higher temperature to be activated. Most importantly, we observe that, at the end of the crystallization process, when the whole volume is totally crystallized, the grains are smaller and more numerous in the N-doped region (see Table 2, 300 °C, 1 h). This characteristic cannot result from heterogeneous nucleation on N-related sites, which would have led to a decrease in the nucleation temperature; therefore, it must be ascribed to the fact that, in the temperature range studied here, the nucleation rate is high while the growth rate is comparatively low. The higher the N concentration, the more pronounced is this effect.

**Figure 5:**
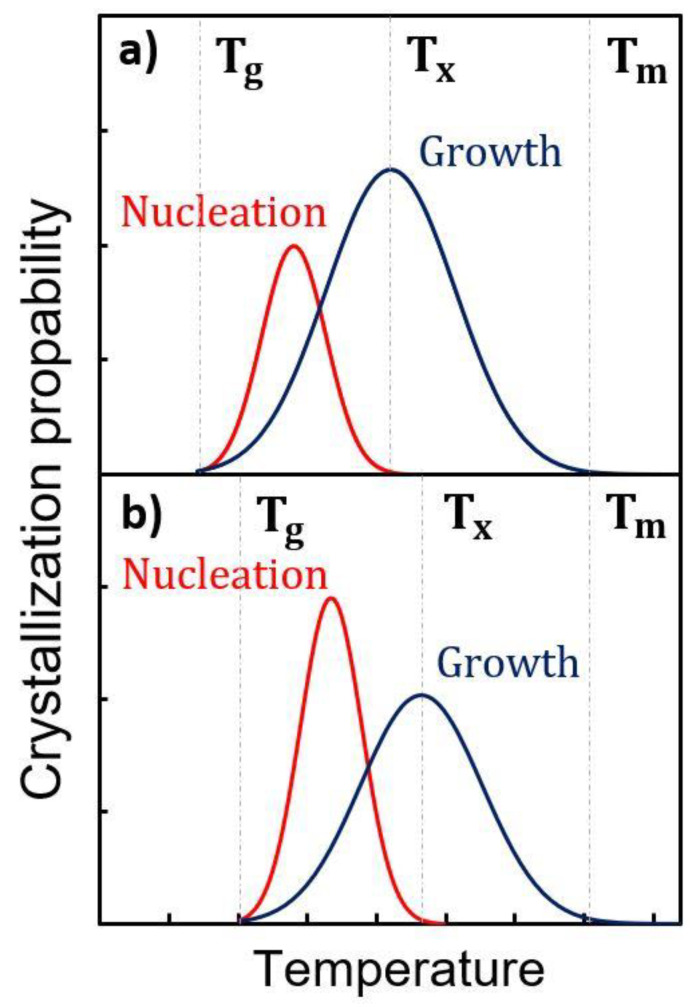
Schematic illustrations of nucleation dominated kinetics for undoped GST-225 (**a**) and N-implanted GST-225 (**b**). T_g_, T_x_ and T_m_ are the glass transition, the crystallization and the melting temperatures, respectively.

In Figure 5, we have schematically compared the crystallization characteristics of undoped and N-doped GST-225. From stress experiments, we know that T_g_, the glass transition temperature, increases with N doping [27]. In contrast, there is no evidence that T_m_, the melting temperature of GST-225, is impacted. There are even reports showing that the Tm of GeTe is unaffected by N doping [40]. We have evidenced that T_x_, the crystallization temperature, is dramatically increased in N-doped GST-225. Thus, T_g_ and T_x_ are shifted in the diagram related to N-doped GST. Actually, from our observations, beyond the shift towards higher temperatures of both the nucleation and growth probabilities, the main effect of N is to increase the ratio between the nucleation and the growth probabilities. This figure illustrates that, in N-doped GST, the crystallization mechanism is more “nucleation dominated” than in pure GST, in the range of temperatures investigated in this work.

From the classical nucleation theory, we understand that this characteristic results from the increase in the viscosity of the amorphous material when doped by nitrogen [3,41,42,43]. This viscosity increase must be related to the mechanically constrained environment of the Ge atoms, some of which are bonded to N [44]. The viscosity of a glass is inversely proportional to the diffusivity of the atomic species that compose it; our results can also be interpreted as being due to the reduction in Ge diffusivity in the presence of N, in the amorphous phase. 

Moreover, the observed resistance of the grain to coalescence should be mentioned. The coalescence of neighboring grains requires the collaborative motion of several tens of thousands of atoms. Again, we can ascribe this resistance to the reduced diffusivity of at least one of the constituents of the material, most probably Ge. However, this occurs in the crystalline phase and, up to now, the exact location of N has remained elusive.

### Strain in the Grains: N Location

A second important question concerns the location of N in the GST material. It is now clear that, in the amorphous phase, N is most probably bound to Ge, which explains the increased viscosity of the material. However, in the crystalline state, the question remains open.

There have been a number of reports evidencing, most often by X-ray photoelectron spectroscopy (XPS), the presence of a Ge nitride phase after the crystallization process [23,32]. The formation of such a phase, sitting preferentially at the grain boundaries (GBs), could eventually explain the reduction in the growth rate. However, if the 5% of nitrogen initially contained in the amorphous material has transformed into Ge_3_N_4_, this phase should be detected. Moreover, the stoichiometric imbalance created by the formation of a second phase involving Ge and N should lead to the formation of Ge-poor GST phases, such as the Ge_1_Sb_2_Te_4_.

Actually, despite our efforts and similarly to Song et al. [45], we have not been able to detect such a phase by EDX or EELS, nor we have noticed any precipitate or thin layer decorating the GBs. We could not evidence the Ge_1_Sb_2_Te_4_ phase either, and all grains show the expected GST-225 cubic or hexagonal structures. On the contrary, the chemical imaging of the crystallized layer with a resolution of about 2 nm suggests that nitrogen is homogeneously dispersed in the grains. If nitrogen, bound to Ge or as molecular N_2_, resides inside the grains, these grains should show some deformation with respect to their regular interplanar spacings. To check this possibility, we have carried out strain measurements, comparing the lattice spacings and characteristic angles in the grains, depending on whether they were found in the N-doped or undoped regions. This situation, where isolated nanocrystals of random orientation are buried in an amorphous matrix and located relatively far from any reference crystal, is well beyond the application fields of all popular strain measurement techniques, working in the image [46,47] or diffraction modes [48,49]. For this reason, we have used the novel AbStrain technique specifically invented to overcome these limitations. Its working principle is described elsewhere [36,50]. We briefly recall the operational procedure which was used. To ensure that the strain that we could eventually evidence mostly resulted from N doping and not from some stress generated by the contact between neighboring grains, we focused our attention on the sample annealed at 210 °C for 30 min, in which the nanocrystals were still growing at the expense of the remaining amorphous matrix. 

First, a reference HR-TEM image of Si lattice was taken along the [110]^Si^ zone axis. This image was used for the measurement of the systematic image calibration errors and distortions arising from the microscope and charge-coupled device (CCD) camera. Secondly, HR-TEM images of many GST crystals were acquired using the same magnification, tilt and defocus conditions, in the unimplanted then in the N^+^-implanted zones, by shifting the sample. Afterwards, the GST crystals showing the fringe contrasts expected from the structure when viewed along the <001>^GST^, <112>^GST^ or <111>^GST^ zone axes were selected for further analysis. After correcting from systematic errors and distortions arising from the microscope, exact measurements of the lattice spacings and angles were carried out (in the Fourier space) on nanocrystals located both in the implanted and unimplanted regions. “Absolute” strain tensor components, i.e., components defined with reference to the perfect bulk GST lattice (as from ICDS file), could then be calculated [36]. 

**Figure 6:**
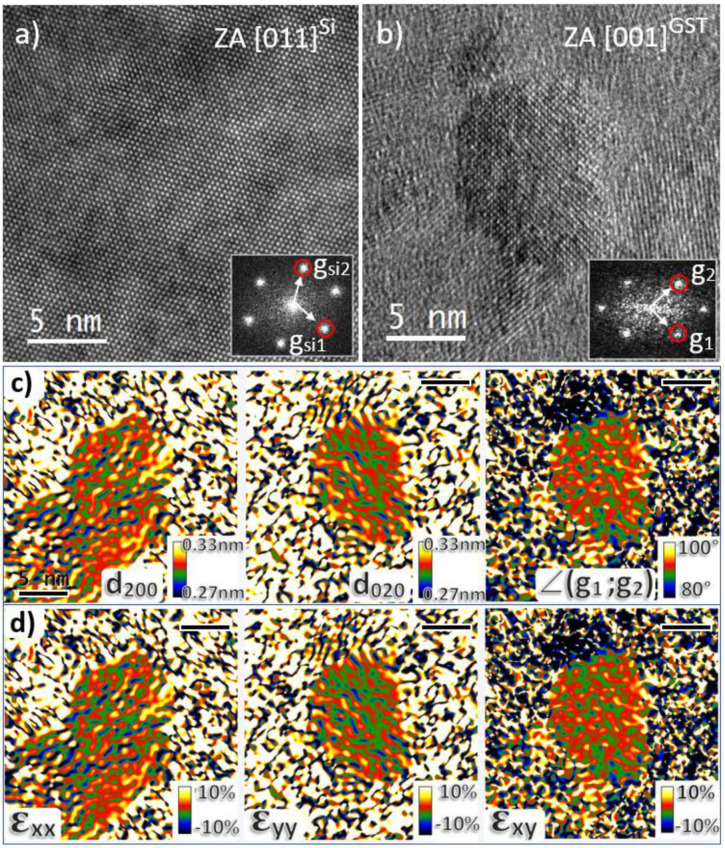
HR-TEM images of the reference Si lattice (**a**) and of a large Ge_2_Sb_2_Te_5_ crystal viewed along the <001>^GST^ zone axis (**b**). Inserts show their corresponding FFTs and the reciprocal vectors used for AbStrain analysis. AbStrain analysis: maps of the (200) and (020) interplanar distances and of the angle between these planes obtained after correction (**c**). Extracted strain components ε_xx_, ε_yy_ and ε_xy_ (reference is the perfect GST-225 cubic lattice) (**d**). The black scale bars refer to a length of 5 nm.

Figure 6 shows an HR-TEM image of the reference Si lattice (Figure 6a), an HR-TEM image of a large Ge_2_Sb_2_Te_5_ crystal located in the unimplanted region and viewed along the <001>^GST^ zone axis (Figure 6b), and the result of the AbStrain analysis of this image (Figure 6c,d). Figure 6c shows the maps of the (200) and (020) interplanar distances and of the angle between these planes. From them, the maps of three strain tensor components, defined with reference to the perfect GST-225 cubic lattice, can be calculated (Figure 6d). The average measured values of d_200_ = 0.302581 nm, d_020_ = 0.301462 nm and ∠(g_1_;g_2_) = 90.7179° are very close to the values characteristic of the relaxed GST lattice expected at d_200_ = 0.301843, d_020_ = 0.301843 and ∠(g_1_;g_2_) = 90°. Consequently, all the three measured strain components were very close to zero. This is a clear evidence that the large nanocrystals located in the unimplanted region of the layer are made of pure and relaxed Ge_2_Sb_2_Te_5_ material. 

**Figure 7:**
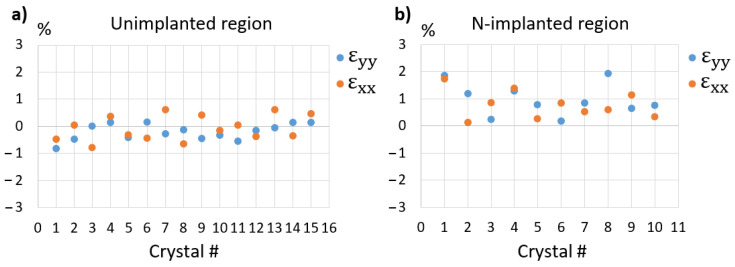
Strain components (ε_xx_, ε_yy_) extracted from 15 large crystals in the unimplanted region (**a**) and 10 smaller crystals in the N-implanted region (**b**). The specimen was annealed at 210 °C for 30 min to initiate crystallization.

The same analysis was carried out on 15 different nanocrystals located in the same region and the results are plotted in Figure 7a. The shear strain components were always close to 0 (<0.05%) and are not shown. The plots of ε_xx_ and ε_yy_ show that their mean values are close to zero (at about −0.1%), and that all measured strain values are below +/−1%, with most of them below +/−0.5%, which must result from the limitations of the technique and from the actual strain states of the nanocrystals. 

Figure 7b plots the results we have obtained on 10 small nanocrystals located in the implanted region. In strong contrast to what was measured in the unimplanted region, large strain values, up to 1.8%, were measured in most of these nanocrystals. Moreover, all these values were found to be positive (mean value: 0.7%), evidence that the nanocrystals are in tension. Clearly, the most probable explanation for this overall positive strain is the presence of N inside the grains, as a bound interstitial or inserted molecule [51]. Whether these N atoms sit between FCC planes or partly fill the vacancies available on one half of the {111} planes would result in the same positive dilatation of the crystalline network and cannot be inferred from our results.

## 5. Conclusions

In conclusion, using specifically designed samples, we have used a combination of in situ and ex situ TEM techniques to study the influence of nitrogen on the thermal crystallization of GST-225. The samples were obtained by nitrogen ion implantation in such a way that a Gaussian-like concentration profile centered at a depth of about 150 nm with a maximum N concentration of 5% was introduced in the 500 nm thick layers. In situ annealing experiments evidenced the need for a higher temperature for nucleating the GST-225 crystalline phase when the N concentration is larger than about 1%. This effect becomes more pronounced as the N concentration increases, with a maximum shift of about 50 °C for 5% of N. Moreover, the growth rate of the grains is limited in the presence of N and crystallization occurs mostly though the nucleation of new grains. As a result, when the material is totally crystallized, grains are smaller and present in larger densities. Thus, these observations show that the effect of N doping is to render the crystallization of the material more “nucleation dominated”. This can be understood as resulting from the binding of N with Ge which, inducing mechanical rigidity to the amorphous network and reducing Ge diffusivity, increases its viscosity [44]. This decrease in Ge diffusivity may also explain the observed resistance of the GST grains to coalescence and to transit from the FCC to the hexagonal phases in the presence of N, during high-temperature annealing.

The location of N in the crystalline phase was quite controversial, and our processors [45] were not able to detect any nitride phase decorating the grain boundaries; therefore, we used a recently invented technique, AbStrain, to measure the lattice spacings of nanocrystals formed by crystallizing N-rich amorphous GST. We have clearly demonstrated that all these nanocrystals are subjected to quite a large positive strain. They are under tension, which strongly suggests that N must be inserted in the lattice.

Globally, all these results demonstrate that the origin of the effect of N on the crystallization of GST-225 is not attributable to the formation of a secondary phase, eventually stable at room temperature, for example a nitride, but to the ability of N to bind to Ge in the amorphous and crystalline phases and to unbind and rebind with Ge along the diffusion path of this atomic species during annealing. 

## Figures and Tables

**Table 1:** Mean Ge_2_Sb_2_Te_5_ grain sizes as functions of annealing temperature.

Temperature (°C)	140	160	170	180	200	230	270	300
Grain Size (±3 nm)	5	10	12	15	18	20	23	30

**Table 2:** Mean GST-225 grain sizes as a function of depth position in the layer and for different annealing temperatures. The BF and DF images used for data extraction are shown in the Supplementary Materials Figure S3.

	Top	Middle (Rp Region)	Bottom	Unimplanted
**175 °C—30 min**	0 (few isolated)	0 (none)	0 (few isolated)	18 nm ± 2 nm
**180 °C—30 min**	6 nm ± 2 nm	2 nm ± 2 nm	6 nm ± 2 nm	20 nm ± 2 nm
**210 °C—30 min**	8 nm ± 2 nm	6 nm ± 2 nm	8 nm ± 2 nm	20 nm ± 2 nm
**250 °C—30 min**	10 nm ± 2 nm	10 nm ± 2 nm	10 nm ± 2 nm	22 nm ± 2 nm
**300 °C—1 h**	10 nm ± 2 nm	10 nm ± 2 nm	10 nm ± 2 nm	>80 nm

## Data Availability

Data available in a publicly accessible repository.

## Supplementary Materials

The following are available online at https://www.mdpi.com/article/10.3390/nano11071729/s1, Video S1: In situ annealing for a pure Ge_2_Sb_2_Te_5_ specimen, taken at 320 °C (25 frame/s, speed × 15), Video S2: In situ annealing for a N-implanted Ge_2_Sb_2_Te_5_ specimen, taken at 140 °C (25 frame/s, speed × 15), Video S3: In situ annealing for a N-implanted Ge_2_Sb_2_Te_5_ specimen, taken at 320 °C (25 frame/s, speed × 15), Video S4: In situ annealing for a N-implanted Ge_2_Sb_2_Te_5_ specimen, taken at 140 °C (25 frame/s, speed × 15), Supplementary Materials, Figure S1 shows the BF image of an as-deposited 500 nm thick Ge_2_Sb_2_Te_5_ specimen, and EDX mapping for the elements present, Supplementary Materials, Figure S2 shows the BF image of a N-implanted Ge_2_Sb_2_Te_5_ specimen, and EDX mapping for the elements present, Supplementary Materials, Figure S3 shows BF–DF images of a N-implanted Ge_2_Sb_2_Te_5_ specimen, annealed at different temperatures and durations (i.e., 175, 180, 210 and 250 for 30 min, and 300 °C for 1 h).
