# Supplementary material for: Effect of Nitrogen Doping on the Crystallization Kinetics of Ge2Sb2Te5
Source: Nanomaterials (Basel). 2021 Jun 30;11(7):1729. doi: 10.3390/nano11071729 (PMC8308197; doi:10.3390/nano11071729)
Supplement: Supplementary file 1 [file nanomaterials-11-01729-s001.zip › Supplementary Materials.pdf]

nanomaterials

Supplementary Materials

Effect of Nitrogen Doping on the Crystallization Kinetics of $Ge_2Sb_2Te_5$

Minh Anh Luong [1], Nikolay Cherkashin [1], Béatrice Pecassou [1], Chiara Sabbione [2], Frédéric Mazen [2] and Alain Claverie [1,*]

[1] CEMES—CNRS, 29 Rue Jeanne Marvig, 31055 Toulouse, France; minh-anh.luong@cemes.fr (M.A.L.); nikolay.cherkashin@cemes.fr (N.C.); beatrice.pecassou@cemes.fr (B.P.)
[2] Léti/CEA, 17 Avenue des Martyrs, F-38000 Grenoble, France; chiara.sabbione@cea.fr (C.S.); frederic.mazen@cea.fr (F.M.)
* Correspondence: alain.claverie@cemes.fr

1. TEM EDX Analyses of the As-Deposited 500 nm Thick $Ge_2Sb_2Te_5$ Sample

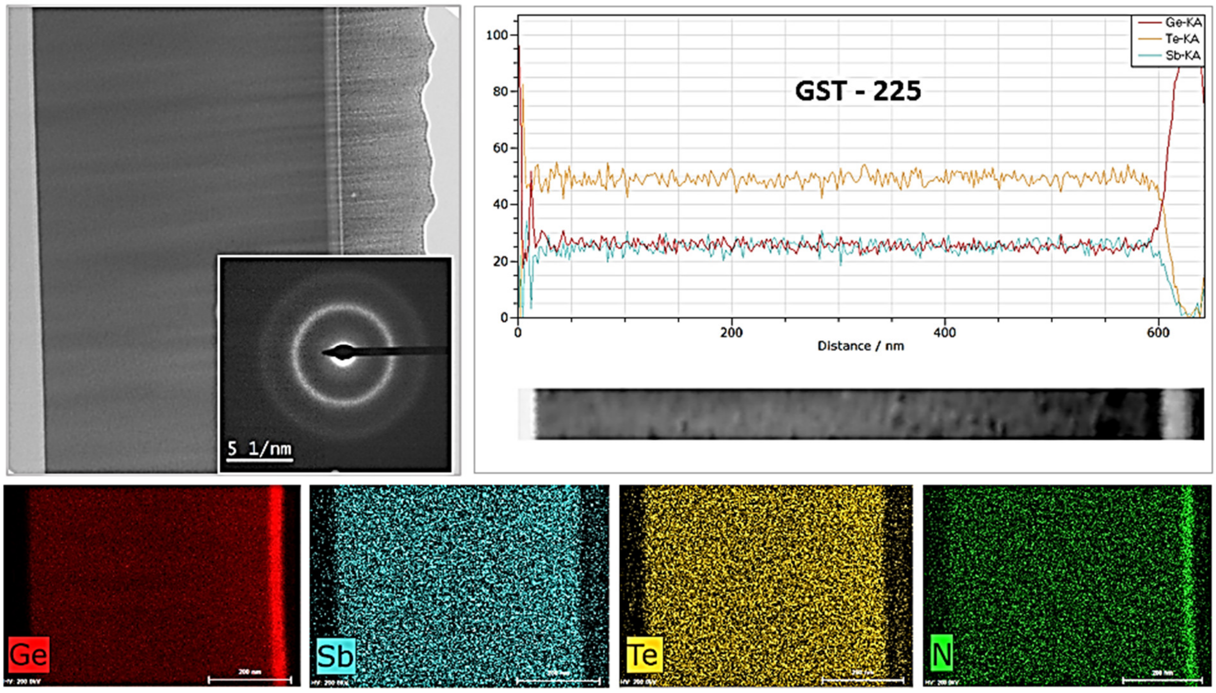

Figure S1. BF-TEM image of the as-deposited 500 nm thick $Ge_2Sb_2Te_5$ specimen and associated chemical mapping obtained by EDX. Ge, Sb, Te and N are displayed in red, blue, yellow and green, respectively.

2. TEM EDX Analyses of the N Implanted $Ge_2Sb_2Te_5$ Sample

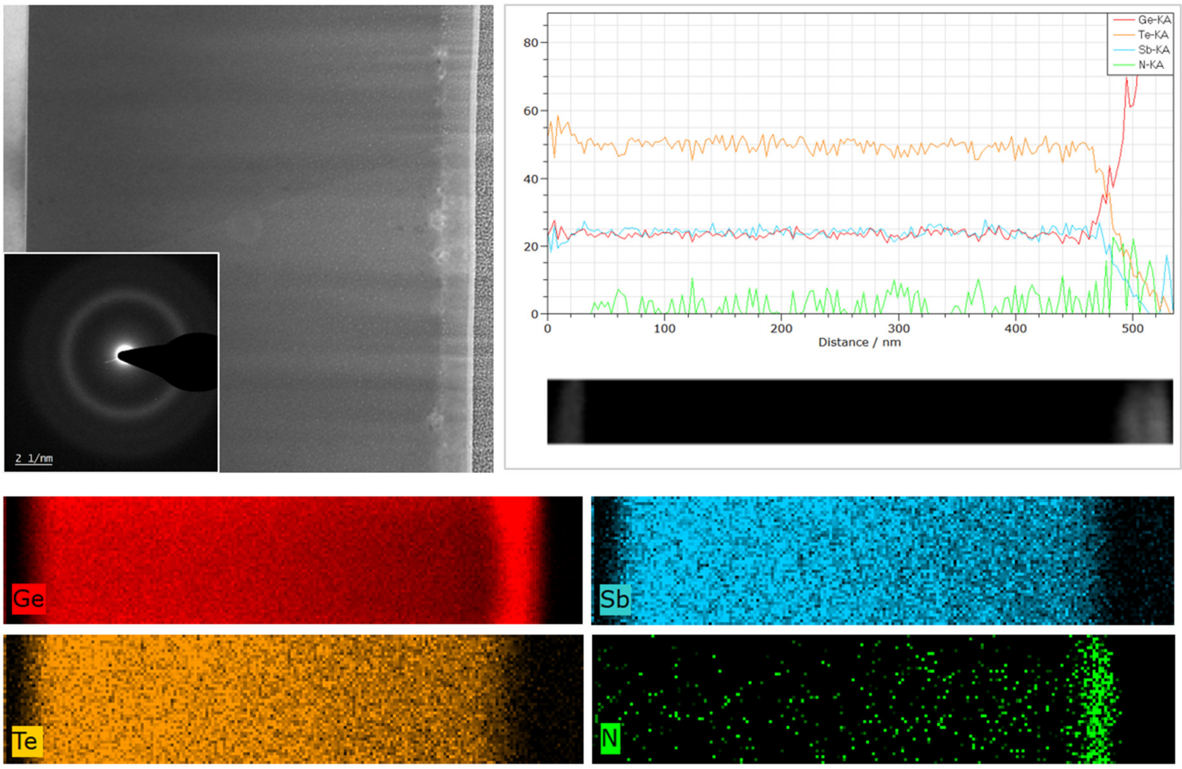

Figure S2. BF-TEM image of the N implanted $Ge_2Sb_2Te_5$ sample and associated chemical mapping obtained by EDX. Ge, Sb, Te and N are displayed in red, blue, yellow and green, respectively. Few voids are seen at the interface between GST and GeN. The EDX line-scan shows traces of N in the implanted region, close to the detection limit.

3. BF and DF TEM Images of the Samples Annealed "ex-situ"at Different Temperatures

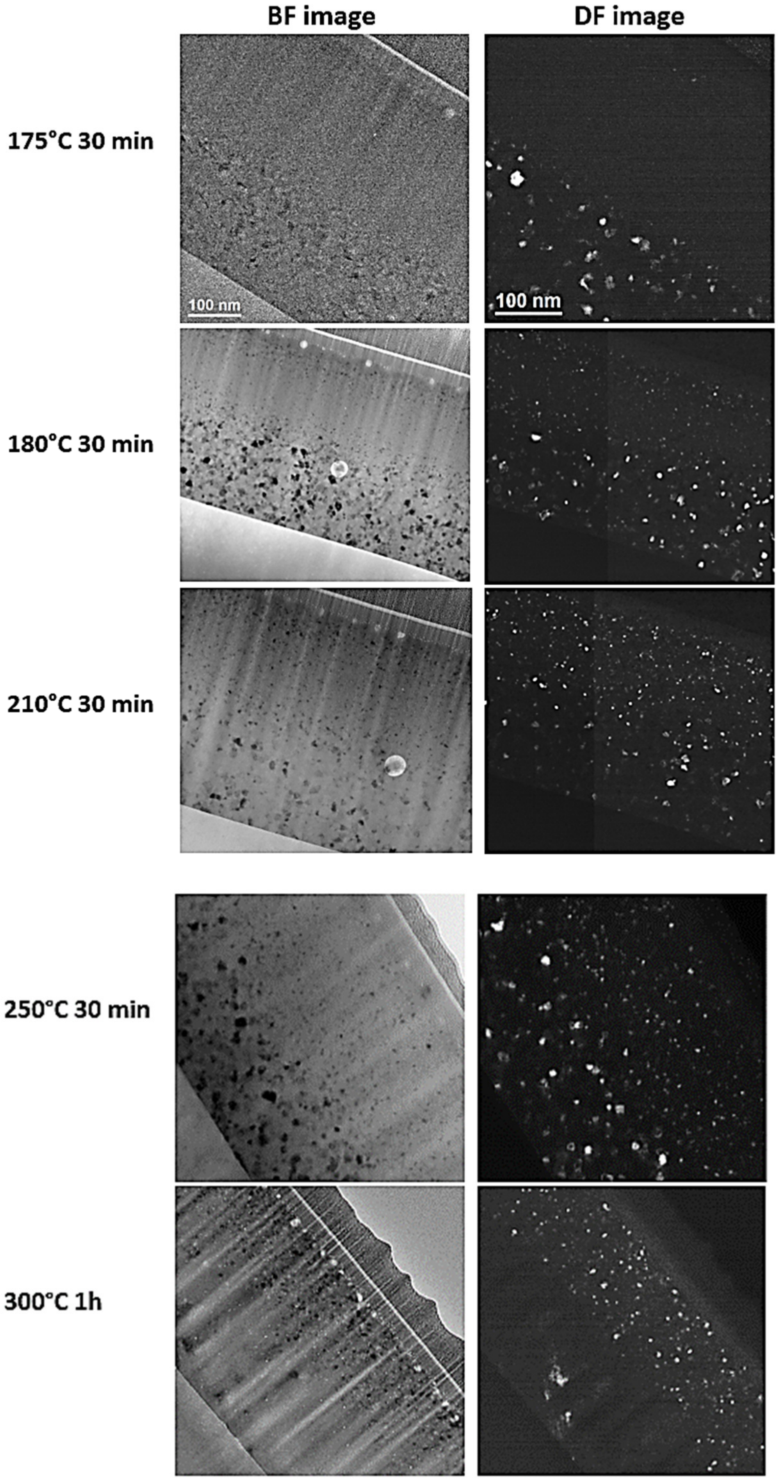

Figure S3. Set of BF/DF TEM images of N implanted $Ge_2Sb_2Te_5$, annealed at different temperatures and durations.
